# Differences in the Effect of Sleep Deprivation on the Postural Stability among Men and Women

**DOI:** 10.3390/ijerph18073796

**Published:** 2021-04-05

**Authors:** Marta Ołpińska-Lischka, Karolina Kujawa, Janusz Maciaszek

**Affiliations:** Department of Physical Activity and Health Promotion Science, Poznan University of Physical Education, 61-871 Poznań, Poland; m.olpinska.lischka@tlen.pl (M.O.-L.); kujawa.karolina5@gmail.com (K.K.)

**Keywords:** sleep deprivation, postural control, posturography, gender difference

## Abstract

Objective: Sleepiness caused by sleep deprivation may increase the risk of injuries and damages during physical activity. Individual data so far indicate a generally better static postural stability of women regardless of sleeping conditions. The main aim of this study was to assess the impact of sleep deprivation on postural stability according to gender after 24 h of sleep deprivation. Methods: Participants included 83 students (36 men and 47 women). Postural stability was measured with eyes open and closed eyes before and after sleep deprivation. Data from posturographic platform were used to assess postural stability objectively. Results: The type of test determined the size of observed changes in postural stability. The data suggest that women are better able to cope with the effects of sleep deprivation than men. Conclusion: Postural control system is very important in sport and in physically active people. The results show that men are more sensitive to sleep deprivation than women because they had higher COP (center of pressure) values in tests. Less postural stability of the body due to sleep deprivation indicates a higher risk of injury during physical activity.

## 1. Introduction

Stable posture is a motor skill that allows to maintain and restore the body balance in any position. Postural stability is a determinant of functional independence and plays an important role in the daily routine. Good stable posture is essential for practicing physical activities. The prerequisite for maintaining a stable posture is primarily the proper functioning of vestibular, somatosensory, and visual systems. The lack of integration of one of these systems may lead to reduced postural stability [1]. In fact, postural control in most activities of daily living, such as standing, is well trained, but for more complicated activities, it requires also other resources to be activated.

Another factor that may affect postural stability is attention and concentration. Nurwulan et al. (2015) found that the dual task, which was based on texting while maintaining balance on the posturographic platform, contributed to lower postural stability in static student conditions [2]. Other studies by Donker et al. (2007) used the paradigm of the dual task in young men and women to study their postural stability using the posturograph. The authors observed that more difficult attempts to say specific words backwards (“Simon”—“nomis”) decreased the postural control [3].

The next important factor affecting the postural stability level is age. Between the ages of 20 and 30, postural stability is the most optimal, because it results from situational experiences [4]. With increasing age, there is a higher probability for developing specific changes. Based on the available results, it can be concluded that postural impairments are more noticeable in older groups compared to younger participants [5,6].

The next factor causing postural instability of the body is a small amount of sleep, which is often characterized by young people [7]. As a result of insufficient sleep, motor fitness, and especially postural body control, deteriorates [8]. Gomez et al. (2008), who applied sleep deprivation to young people, noticed a deterioration in postural stability after just 24 h of sleep deprivation, but only in a task with closed eyes on a vibrating platform. Unexpectedly, after 36 h of sleep deprivation, there was no further deterioration in postural stability, and the number of swaying was similar to that after 24 h of sleep deprivation [9]. In several cases, even less swaying was recorded after 36 h of sleep deprivation than after 24 h of sleep deprivation. However, in the study conducted by Nakano et al. (2001), it was observed that 19-h sleep deprivation negatively affected postural control in men, and swaying was greater in the sample with closed eyes than in the sample with open eyes [10].

The level of postural control may also vary between genders [11]. Fewer gender differences are observed among young adult women and men, but the results obtained so far are inconsistent. Gender differences in postural control have sometimes been reported under normal sleep patterns. During 60-s posturographic tests with closed and open eyes, the female sex recorded better static stability [8]. In the study by Greve et al. (2013), young women and young men aged 20–27 years were examined with two 20-s static measurements separated by one-minute intervals. Postural stability indicators were better in women than in men in the open-eye study [12]. However, the assessment of postural stability under conditions of relative silence with closed and open eyes among PE students clearly was in favor of women compared to men [13]. There are also some recent studies that indicate better values of posturographic parameters of women, although the difference in these studies was small [14]. Gender-related differences in the values of stability parameter were also observed [15]. The authors reported better static postural stability of female soldiers in the eyes open and eyes closed conditions compared to men. Further studies have shown better postural stability in men. Blaszczyk et al. (2014) investigated balance performance in a group of healthy students of physical education. In this study, women had poorer postural stability (higher center of mass (COM) and center of pressure (COP) velocities) compared to men [16]. In another study, women had also significantly worse postural control than men [17].

There are also studies available where no differences in postural stability between women and men have been shown [18]. In the Bryant et al. study (2005), there were no differences between men and women in the posturographic study during static samples [19]. Lee and Petrofsky (2018) even studied differences in postural control in men and women during menstruation and ovulation. There were no differences in postural control between women during menstruation and men. Significant statistical changes were observed only in women during ovulation, where men showed less body swaying in the position with their feet apart and eyes closed on a hard surface and in the three most difficult tasks. The authors suggest that increased limpness in the main ligaments of the foot occurs during ovulation, and this may cause more frequent sports injuries in women during ovulation [20].

Therefore, gender and sleep deprivation are factors that may affect postural stability [1]. Several studies are available that describe postural stability after sleep deprivation. However, postural stability is usually assessed after a normal sleepy night. In addition, the majority of studies in this area have been conducted mainly among people with visual impairment [21] and other clinical populations such as Parkinson’s [22] and multiple sclerosis [23]. The results of studies in such populations usually provide more obvious and predictable results, but the mechanism of these changes may be more determined by the disease than by sleep deprivation. There is a lack of studies among young and healthy people, and therefore, studies in young people with impaired sleep deprivation assessing gender differences in posture control seem to be necessary to analyze the differences and mechanisms of their development. Sleepiness caused by sleep deprivation may increase the risk of injuries and damages during physical activity. However, physically active people often have better tolerance to sleep restriction, which is why they often show less negative effects due to worse sleep quality than physically inactive people [24]. In addition, although individual data so far indicate a generally better static postural stability of women regardless of sleeping conditions, the data are insufficient. Therefore, the main aim of this study is to assess the impact of sleep deprivation on postural stability according to gender after 24 h of sleep deprivation.

On the basis of literature review, two hypotheses were formulated:

1. COP path length will be extended after sleep deprivation, regardless of the role of sight in maintaining postural stability.
2. Sleep deprivation will negatively affect the postural stability level, increasing the COP path length more in men than in women.

## 2. Materials and Methods

### 2.1. Ethics Statement

All participants gave their informed written consent prior to participation according to procedures approved by the Ethics Committee of the Poznan University of Medical Sciences in Poland, approval number 989/17.

### 2.2. Sample and Recruitment

The study population was recruited after the lectures from the University of Physical Education. The objectives and procedures of the study were explained to all subjects. Students were told that the study involved testing postural control after 24 h of sleep deprivation, but no information was given regarding the anticipated results. The students were informed that the study consists of three sessions (zero test, 1st session, 2nd session). The subjects were informed by the researcher about the detailed course of the experiment. Then, the participants signed the informed consent form. 

The experiment was conducted among students of physical education studies. The study included 107 students. Of the 107 subjects who were enrolled in the study, 24 subjects withdrew due to night work (*n* = 2), orthopedic problems (*n* = 5), complications without precise definition (*n* = 17). A priori power calculations were conducted with G*Power 3.1.9.7. (free software: http://www.gpower.hhu.de/en.html, HHU Düsseldorf, Germany) [25] to determine sample size. This analysis was based on an assumed effect size of 0.3 (medium to large), alpha error of 0.05, and power of 0.80. We found that the required number of participants in this study was 68 subjects.

A total of 83 young, healthy people took part in the experiment. The students were divided into two groups. The experimental group (EG) consisted of 25 women (Mean_age_ 21 ± 1.04 SD_age_) and 17 men (Mean_age_ 21.36 ± 1.01 SD_age_), and the control group (CG) consisted of 22 women (Mean_age_ 21.63 ± 0.78 SD_age_) and 19 men (Mean_age_ 21.58 ± SD_age_ 0.04 years). The demographic characteristics are presented in Table 1.

The number of men and women differed in each group due to the period in which the research was conducted. Most of the students have internships at that time, which made it impossible for us to gather an equal number of subjects in both groups. The most important criteria for selecting the sample was ‘’good’’ to ‘’very good sleepers’’, which was subjectively assessed on the basis of a self-designed questionnaire. Since willing participants met these criteria, they were not randomly assigned to groups. The examined students determined the preferences to which group they want to be assigned. The number of subjects was considered sufficient [26,27,28]. 

The study included persons who met the following inclusion criteria. The inclusion criteria were as follows: abandonment of alcoholic and caffeine beverages on the day of study, consumption of the last meal before 8 pm not exceeding 350 kcal and consent to participate in the study. The exclusion criteria were also included age between 18 and 30 years; use of sleeping pills, mental illness and acute medical conditions, no injuries on the legs, and no history of balance or orthopedic problems based on self-reports. During the qualification interview, the participants were informed about the scope of the study and the possibility of resigning from participation in the experiment at each stage without giving any reason.

### 2.3. Measure

During the initial measurement period, the subjects received a self-designed questionnaire to complete. The questionnaire consisted of 12 questions about sleep duration during the previous month, taking naps, use of sleeping medication, assessment of physical activity, and coffee/alcohol consumption in late afternoon/evening hours. 

Throughout the first session, participants’ anthropometric characteristics were carried out (height, weight, body mass index (BMI)). During the measurements (1st session, 2nd session), the room was silent, and the room temperature was comfortable for the participant’s attire. No noise was perceived that could alter proper data collection. Only two researchers and the participant were present in the room.

To estimate postural stability, COP data were collected using the force platform (AMPI PJB-101 model, AMTI, Waterdown, MA, USA). The subjects stood barefoot to ensure footwear had no effect on the postural stability. A researcher instructed each participant to stand in a relaxed position while looking forward and keeping their arms relaxed at their sides.

Posturographic tests were carried out on the AccuGait AMPI PJB-101, Waterdown, MA, USA platform. The sequence of tests was the same for each participant in the experiment. They have to perform two tasks in the same order. The duration of a single test during static stance was 30 s, which is an appropriate time to record a reliable COP measure [29] The following measurements were conducted: measurement with eyes open in free position (EO), measurement with eyes closed in free position (EC). A week later, an identical measurement session took place. In addition, the Romberg index was calculated from these tests. Values that are higher than 1 indicate greater oscillation with the eyes closed. Zero or negative values mean lower postural oscillation with the eyes closed [30]. 

Among the parameters obtained from our own observations and literature analysis, it was assumed that path length [31], sway area, and sway velocity [32] are the most valuable for estimating postural stability. Therefore, the most commonly used parameter for analysis was COP path length, which determines the distance traveled by COP (mm). The path length is known to be a reliable and valid measure of standing balance [31]. A shorter path length means better postural stability. The second metric that we used is sway area, and it is defined as the area of the 95% confidence ellipse around the COP trajectory (mm^2^). This metric allows assessing the size of the area of the COP movement on a force plate. A smaller area surface means a better performance [33]. Velocity was not calculated due to the strong correlation of the parameter with path length [34,35].

There are a large number of parameters in stabilometry. Many of them can often give irrelevant and random results, which can be misleading [36]. Therefore, we examined two parameters that are considered sensitive with high reliability.

### 2.4. Procedure

One week before each testing (1st and 2nd session), the subjects had to maintain regular sleep schedules. Participants were advised to perform normal daily activities. No intake of caffeine, alcohol, energy drinks, and late physical activity were allowed before each session.

The experiment consisted of three sessions for each group. Each student participated in initial measurements (zero test), which took place a few days earlier and were aimed at familiarization with the tests.

All subjects were tested two times for each group. There were no differences observed between the 1st session for all groups. Both groups were tested after a night’s sleep (CG: 1st session: non-sleep deprivation, EG: 1st session: non-sleep deprivation). The second measurement differed between the studied groups. The control group was examined after a normal night’s sleep (CG: session 2nd: non-sleep deprivation), so the control group was given the opportunity for full sleep. The EG remained awake and was monitored by researchers at the laboratory throughout the night (EG: session 2nd: sleep deprivation).

Testing procedures were separated for one week. All measurements were taken between 6:30 am and 8 am due to the possible influence of time-of-day on postural control [37]. After finishing all testing sessions, participants returned to their daily activities, including study. 

On the day of study, in the morning, the experimental group received an actigraph (Caltrac, Muscle Dynamics, Inc., Tarrance, CA, USA) to measure their daily physical activity. It was fixed with a belt to the stomach level. Subjects (EG) wore the Caltrac for 24 h. The participants were required not to take naps during the day, which was verified by a daily physical activity measurement using actigraphs. The experimental group received the actigraphs on the day of the experiment (8:00 am). The results were not presented in the text, because the measurement only had a control purpose to check whether the study participants were physically active during the day.

The experimental group spent the sleepless night in the university building under the care and control of the researchers. To ensure the testing procedure was in accordance with the protocol, and participants were monitored under the supervision of a research staff throughout the night. During the test, activating forms were planned for the participants, such as board games, playing cards, puzzles. Such activities as watching videos of strong emotional valence or playing computer games were prohibited. Before the main study after sleep deprivation, students could have breakfast and drink water.

### 2.5. Statistical Analysis

For statistical evaluation, Statistica 10 software (StatSoft Inc., Tulsa, OK, USA) was used. A three-way analysis of variance Analysis of variance (ANOVA) was used to compare groups (control, experimental), gender (men, women), and conditions (sleep deprived and sleep non-deprived). Tukey’s post-hoc test was implemented for pairwise comparisons.

The effect of sleep deprivation in the control and experimental group was evaluated within the COP_path length_ and COP_sway area_ parameter in the sample with open and closed eyes, and then the Romberg index was calculated with the following formula: [(surface EC/surface EO)]. Main effects and interaction effects in the studied groups were determined. Differences between control and experimental groups were explored using the Mann–Whitney U-rank test. In order to characterize the collected material, the basic measures of descriptive statistics were calculated and presented using the arithmetic mean (Mean) and standard deviation (SD). Partial eta squared (η2) was used as an effect size indicator. Statistical significance was determined at the level of *p* < 0.05.

## 3. Results

Subjective sleep characteristics were assessed with self-designed questionnaire. Analysis shows that the largest percentages of respondents were ‘’good sleepers’’ (61%) followed by ‘’very good sleepers’’ (39%). Overall, the mean duration of sleep for female participants was shorter (6.70 ± 1.86 h) compared to male students (7.12 ± 0.76 h) during a typical school week. More than half of female students took naps during the day (women: 52%, men: 44%). It was found that none of the students took any sleeping pills. Overall, most of the students undertook physical activity in late afternoon/evening hours (women: 78%, men 90%). A higher percentage of women (31%) compared to men (22%) consumed coffee in late afternoon/evening hours.

Analysis of the results with ANOVA test shows the size of changes in mean values for the parameter COP_path length_ and COP_area_ in standing position.

The effect of group. Non-significant differences were observed between the groups in COP_path length_ (eyes opened: F_(1,77)_ = 0.53, *p* = 0.467, ɳ2 = 0.038; eyes closed: F_(1,73)_ = 0.26, *p* = 0.606, ɳ2 = 0.003). In the COP_area_, the effect of group was also not significant (eyes opened: F_(1,63)_ = 1.68, *p* = 0.199, ɳ2 = 0.026; eyes closed: F_(1,63)_ = 0.62, *p* = 0.432, ɳ2 = 0.008) (Table 2).

**Figure 1:**
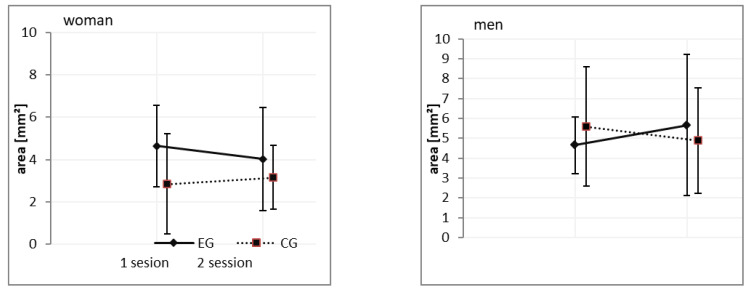
Gender related differences in COP_area_ under eye open conditions after 24-h sleep deprivation. Note: EG: experimental group; CG: control group.

The effect of gender. For COP_area_ (EO), a significant main effect of the gender was identified (F_(1,63)_ = 10.24, *p* < 0.01, ɳ2 = 0.139). The results indicated that the quiet standing condition showed a smaller area in women then in men in both sessions (Table 2, Figure 1).

**Figure 2:**
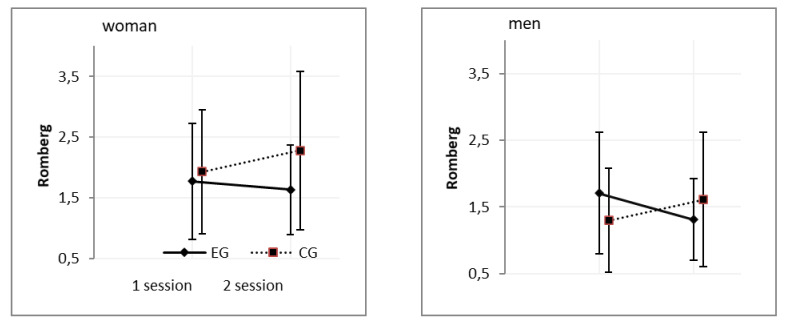
Romberg ratio for the COP_area_ after 24 h sleep deprivation in women and men. Note: EG: experimental group; CG: control group.

A comparison of obtained Romberg indices for the COP_area_ parameter revealed a statistically significant main gender effect (F_(5,88),_ *p* < 0.05; ɳ2 = 0,085). However, the η2 value was weak (Table 2, Figure 2).

**Figure 3:**
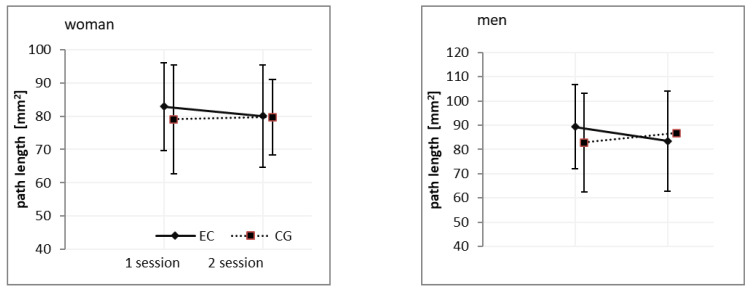
Gender related differences in COP_path length_ under eye close conditions after 24-h sleep deprivation. Note: EG: experimental group; CG: control group.

Interaction. There was a statistically significant interaction effect of the session*group in COP _path length_ (EC) (F_(1,73)_ = 4.28; *p* < 0.05, ɳ2 = 0.055). However, post-hoc tests (Tukey) showed no significant differences in means at different factor levels (Table 2, Figure 3).

In addition, in the COP_area_ (EC), a statistically significant effect of the interaction was found session*group (F_(1.63)_ = 8.72, *p* < 0.01, ɳ2 = 0.12). In the experimental group, the COP_area_ (EC) level was higher in the 1st session than in the 2nd session. In other cases, the differences were not statistically significant (Table 2).

## 4. Discussion

It was found that 24-h sleep deprivation did not adversely affect the posture control of examined women. In men, the results after sleep deprivation showed a deterioration in the post urography parameters. 

Stable posture depends largely on visual stimuli. Our first measurement was conducted in a calm standing position with open eyes. Pham et al. (2014) believe that such a simple test is not enough to expect any changes in postural control after sleep deprivation [38]. However, in other studies [26,39] and in our own study, this test constituted a reference point for the rest of the tests. Usually, the average values of open eye stabilometric parameters are often lower than in other tests. This shows that this measurement is correctly used for comparison with other tests. The sway area values in EO conditions were lower than in the EC conditions in both female and male groups regardless of the test session. In the experimental group of men, higher sway area values (1st and 2nd session) were noted than in women. An increase in the COP path length in the open eye test after sleep deprivation was only recorded in men in the experimental group, and this indicates less stable postural control of men after a sleepless night. The mechanism of change is unclear, but postural stability is strongly associated with lower limb muscle strength [40] and requires active modulation of muscle activity even in a standing position [41]. Sleep restriction can weaken muscle strength [42]. The effects of sleep deprivation are comparable to those associated with poor sleep quality. Chen et al. (2017) found that shorter sleep duration was a factor in lowering muscle strength in students, but the impact of poor sleep quality on muscle strength is not clear [43]. The study conducted by Kujawa et al. (2020) showed that 24 h sleep deprivation reduced lower limb muscle strength in young students [24], and this may have been due to a change in body temperature after 24h sleep deprivation [44]. In turn, this may have resulted in a decrease in muscle function [10]. Postural instability caused by sleep deprivation is probably correlated with this factor. Identifying possible changes in muscle strength after sleep deprivation may be difficult due to the changes already occurring in the muscles [24]. However, these correlations require further studies with EMG.

Visual information may lead to both abnormal postural control of the body and its improvement [45]. In our study, the elimination of visual control resulted in higher COP path length values compared to the open eye study in the experimental and control group. However, based on a literature review [6,26,46], postural stability was expected to deteriorate after 24-h sleep deprivation in the studied students. However, in the closed-eye sample, the COP_path length_ recorded after sleep deprivation did not deteriorate in the experimental group of men and women. Unfortunately, due to the lack of such analyses of stability changes in sleep deprivation according to gender, our observations cannot be compared with others. It is difficult to explain the reason for the lack of deterioration of body stability after sleep deprivation in the experimental group in this study. It is known that postural control is strongly related to attention processes [46], and cognitive load has an important role in postural control in sleep deprivation [47]. Sleep deprivation impairs cognitive functions and leads to a decrease in regional brain activity. The thalamus and prefrontal cortex regulate cognitive functions such as vigilance and attention. Due to increased sleepiness, tasks requiring more attention become more difficult to perform and require more effort [48]. However, healthy and young people are characterized by better cognitive processes than older people [49], and proper focus of attention can even improve posture control after sleep deprivation [50]. It can be assumed that students, despite the lack of visual control, were able to concentrate in such a way that the lack of sleep did not negatively affect their posture stability. On the one hand, a small number of simple tests can temporarily increase the attention and motivation of test subjects while suppressing the effects of sleep deprivation [51], and the tasks perceived as difficult can lead to reduced motivation [52]. This assumption may explain our results in part. It seems necessary to test this effect by applying longer trials and longer sleep deprivation among young people, which will increase their level of sleepiness, and this may lower the motivation to consciously control posture. This potential explanation, although speculative due to the difficulty of measuring, is possible.

In addition, other studies [53] noted that the subjects were more alert in standing position than sitting after 28 h of sleep deprivation. The EEG measurement showed that the vertical position increased excitement and allowed them to maintain attention. This may be another aspect explaining the results. 

Lastly, it cannot be ruled out that the physical activity of the participants might have influenced the results. The role of physical activity in improving postural stability is probably large. Perhaps, it was a factor in which postural stability did not deteriorate in some tests after 24 h sleep deprivation. Coco et al. [54] demonstrated in their study that several of the negative effects of sleep deprivation can be reduced through regular physical activity. In the study, 72 h of sleep deprivation was used in an endurance athlete, and it was proved that his mental and physical well-being did not deteriorate after sleep loss. Therefore, it would be worthwhile to investigate the gender differences using a specially designed study needed to clarify this issue, especially that the positive effect of exercise is noticeable in men compared to women [55].

It is also worth emphasizing the possible influence of sports shoes on the level of postural stability. It has been confirmed that specialist footwear with stiff ankle support can permanently change the mobility of the ankle in barefoot standing conditions, which is associated with reduced postural stability [56]. This factor should be taken into account in the next experiment, due to the high physical activity of the students resulting from the nature of the studies. 

Considering the approaches mentioned above, this means that many aspects should be examined. It is certainly not simple to determine in detail the role of individual factors due to the wide scope of research, but the elucidation of individual relationships is important in order to identify changes in postural stability due to sleep deprivation.

The Romberg test is used to compare body imbalance in positions with open and closed eyes [57]. Many studies have been conducted using this test in conditions after a normal night’s sleep, but only a few studies have used sleep deprivation to test postural control. After sleep deprivation, Ma et al. (2009) did not report significant changes in (male) students’ (Romberg’s) postural control [39]. In another study, Bougard et al. (2019) found that the Romberg index values were higher in the closed-eye test, but they did not deteriorate after sleep deprivation [27]. In our experiment, the analysis of changes in the Romberg _path lenght_ index values showed a higher sensitivity to visual stimuli in the male group than in the female group after sleep deprivation (*p* < 0.05). This means that men are more sensitive to a lack of visual information, which affects their less stable body posture. Statistical significance was not found in the value of the Romberg_area_ coefficient parameter in men. This can be explained by the different nature of these two indicators. The path length parameter determines the changes originating in the proprioceptive and motor systems [58], and the sway area parameter results from the vestibular function [59].

Collins and DeLuca (1995) put forward a hypothesis that the lack of visual stimuli increases the rigidity of the musculoskeletal system, which leads to a destabilization of postural control [60], and this could be the reason for greater changes in postural stability in the male group. In women, no statistical significance was found in the Romberg coefficient. A smaller area of swaying posture in a static position may indicate increased muscle tension causing postural immobilization [61]. Summarizing further physiological studies between muscle activity and COP displacement are needed to confirm this assumption [62]. Since the obtained Romberg’s coefficient values cannot be referred to any other publication, which is based on a similar test procedure assessing gender differences in detail, further measurements (in other study groups) are needed to confirm the recorded results.

Disrupted sleep is a problem, with numerous contributing factors. The results of our research show that sleep deprivation has physical consequences that negatively affect the functioning of a person in everyday life. In addition to less precise motor control, there are other effects, such as hormonal impairments. For example, an inadequate amount of sleep results in decreased insulin sensitivity and can alter glucose homeostasis, which can contribute to weight gain [63].

Insufficient sleep can also affect behavioral effects, which very often result in taking up risky behaviors, e.g., substance abuse, increased food intake, illicit drug use, texting while driving). Such behavior may be the result of a misjudgment of the situation caused by insufficient sensory information and the desire to achieve immediate goals without considering all aspects of a problem [64].

### 4.1. Practical Implications

The results of this study have practical implications for the assessment of postural stability after 24 h of sleep deprivation in physically active people and athletes. However, these results should be interpreted in a proper context.

First of all, lack of sleep is a frequent phenomenon, especially among students due to their very active lifestyle (studying and working at the same time), so building awareness of the consequences of sleep deprivation is necessary.

Secondly, students of physical education are often involved in various types of sports by taking part in competitions. The use of an objective tool for the assessment of postural stability by the coaches in sports clubs can help to identify less effective people, thus reducing the risk of injuries and lower limb injuries among trainees.

Although not all aspects have been clarified, we believe that the results presented in this study make an important contribution to the study of the effects of sleep deprivation on postural stability and may constitute an important step in the development of future research in this direction. We believe that these results could serve as a basis for the development of gender-specific risk assessment tools to effectively prevent lower limb injuries.

### 4.2. Limitations

As previously mentioned, it may be more difficult to detect gender differences in healthy people than in elderly or dysfunctional population. Therefore, the tests should have been more detailed. For example, Persiani et al. (2015) observed intergender differences in their research during optic flow performance [65].

The authors suggested that optic flow stimulation causes asymmetry in postural balance to maintain the control of posture. In addition, the significance of limb loading was indicated, showing that women equally burdened their limbs compared to men, which could be due to the type of physical activity undertaken. It is possible that the use of such attempts will allow more complex conclusions to be drawn.

Some papers reported a greater variability in postural stability throughout the day [40], which was still present on the day after the sleep reduction in subjects. However, repeated postural control studies in other papers showed inconsistent results [28]. Due to the postural variability suggested by the above-mentioned researchers, depending on the time of day, it is worthwhile to carry out measurements more often during the whole day, especially in the case of healthy people. Our study was conducted in the morning hours, and the measurement was conducted only once at each of session. Since other parameters were analyzed at the same time, it would be tiresome and tedious for the subjects to test postural stability several times at two measurement sessions. This could be a factor determining the results obtained in subsequent measurements. It is possible that several measurements of postural control during the day after sleep deprivation would show greater differences between men and women.

In addition, when measuring with open and closed eyes (EO, EC), it was noticed that the mean COP path length results of the control group women between sessions did not deteriorate significantly (*p* > 0.05). Such a result may indicate differences in the quality of sleep-in women. Therefore, sleep quality should be assessed before and during the entire experiment to confirm if the same pattern of changes for EO and EC conditions in each group exists.

## 5. Conclusions

In conclusion, it was found that sleep deprivation did not affect the deterioration of postural control in all the tests. The type of test (with open eyes or closed eyes) determined the size of observed changes in postural stability. Therefore, the first hypothesis should be rejected.

The data suggest that women are better able to cope with the effects of sleep deprivation than men. This confirms the second hypothesis that young female students show more stable body posture than men after sleep deprivation. 

Postural control system is very important in sport and in physically active people. The results show that men are more sensitive to sleep deprivation than women because they had higher COP path length values in tests. Less postural stability of the body due to sleep deprivation indicates a higher risk of injury during physical activity.

## Figures and Tables

**Table 1:** Subjects’ demographic characteristics.

Demographic Characteristic	Men			
	CG		EG	
	Mean	SD	Mean	SD
Age	21.58	0.04	21.36	1.01
Height (cm)	1.81	0.06	1.82	0.04
Weight (kg)	78.38	11.21	79.28	6.57
BMI	23.77	2.54	23.89	1.89
**Demographic Characteristic**	**Women**			
	**CG**		**EG**	
	**Mean**	**SD**	**Mean**	**SD**
Age	21.63	0.78	21.48	1.04
Height (cm)	1.67	0.03	1.69	0.06
Weight (kg)	63.66	6.81	64.16	7.01
BMI	22.71	2.22	22.22	1.95

**Table 2:** Changes in the sway area and path length of postural tests before and after sleep deprivation in the control and experimental group.

	Men		Women		
Area	CG	EG	CG	EG	Interaction
	Mean ± SD	Mean ± SD	Mean ± SD	Mean ± SD	F(*p*)
EO ^1^ [mm^2^]	5.58 ± 2.99	4.64 ± 1.44	2.85 ± 2.35	4.63 ± 1.91	F(1.63) = 3.19, *p* = 0,07
EO ^2^ [mm^2^]	4.89 ± 2.64	5.66 ± 3.53	3.16 ± 1.50	4.02 ± 2.43	
EC ^1^ [mm^2^]	6.40 ± 3.34	7.47 ± 3.79	4.84 ± 3.96	7.53 ± 4.21	F(1.63) = 1.22, *p* = 0.27
EC ^2^ [mm^2^]	6.76 ± 2.87	6.26 ± 2.82	6.44 ± 3.50	5.69 ± 3.22	
Romberg ^1^	1.29 ± 0.78	1.70 ± 0.91	1.93 ± 1.02	1.77 ± 0.94	F(1.63) = 0.12, *p* = 0.72
Romberg ^2^	1.61 ± 1.01	1.31 ± 0.61	2.28 ± 1.30	1.63 ± 0.74	
	**Men**		**Women**		
**Path Lenght**	**CG**	**EG**	**CG**	**EG**	**Interaction**
	**Mean ± SD**	**Mean ± SD**	**Mean ± SD**	**Mean ± SD**	**F(*p*)**
EO ^1^ [mm^2^]	68.79 ± 8.20	66.95 ± 8.29	64.48 ± 6.88	68.91 ± 8.00	F(1.77) = 0.29, *p* = 0.58
EO ^2^ [mm^2^]	71.16 ± 8.46	70.54 ± 12.01	64.00 ± 6.16	67.32 ± 13.29	
EC ^1^ [mm^2^]	82.81 ± 20.40	89.36 ± 17.30	79.06 ± 16.28	82.83 ± 13.24	F(1.73) = 0.99, *p* = 0.32
EC ^2^ [mm^2^]	86.81 ± 20.30	83.43 ± 20.58	79.68 ± 11.35	80.00 ± 15.26	
Romberg ^1^	1.20 ± 0.26	1.34 ± 0.21	1.22 ± 0.20	1.20 ± 0.17	F(1.73) = 2.88, *p* = 0.09
Romberg ^2^	1.21 ± 0.25	1.16 ± 0.18	1.24 ± 0.13	1.21 ± 0.22	

^1,2^—1st session, 2nd session, CG—control group, EG—experimental group, interaction F(*p*)—session*group*gender.

## Data Availability

The data supporting reported results are available in the corresponding author.

## Abbreviations

EG	experimental group
CG	control group
EO	eyes opened
EC	eyes closed
